# Addressing dyslipidemic risk beyond LDL-cholesterol

**DOI:** 10.1172/JCI148559

**Published:** 2022-01-04

**Authors:** Alan R. Tall, David G. Thomas, Ainara G. Gonzalez-Cabodevilla, Ira J. Goldberg

**Affiliations:** 1 Division of Molecular Medicine, Department of Medicine, Columbia University, New York, New York, USA.; 2 Division of Endocrinology, Diabetes and Metabolism, Department of Medicine, New York University Grossman School of Medicine, New York, New York, USA.

## Abstract

Despite the success of LDL-lowering drugs in reducing cardiovascular disease (CVD), there remains a large burden of residual disease due in part to persistent dyslipidemia characterized by elevated levels of triglyceride-rich lipoproteins (TRLs) and reduced levels of HDL. This form of dyslipidemia is increasing globally as a result of the rising prevalence of obesity and metabolic syndrome. Accumulating evidence suggests that impaired hepatic clearance of cholesterol-rich TRL remnants leads to their accumulation in arteries, promoting foam cell formation and inflammation. Low levels of HDL may associate with reduced cholesterol efflux from foam cells, aggravating atherosclerosis. While fibrates and fish oils reduce TRL, they have not been uniformly successful in reducing CVD, and there is a large unmet need for new approaches to reduce remnants and CVD. Rare genetic variants that lower triglyceride levels via activation of lipolysis and associate with reduced CVD suggest new approaches to treating dyslipidemia. Apolipoprotein C3 (APOC3) and angiopoietin-like 3 (ANGPTL3) have emerged as targets for inhibition by antibody, antisense, or RNAi approaches. Inhibition of either molecule lowers TRL but respectively raises or lowers HDL levels. Large clinical trials of such agents in patients with high CVD risk and elevated levels of TRL will be required to demonstrate efficacy of these approaches.

## Introduction

Imagine a 60-year-old patient with metabolic syndrome (obesity, hypertension, insulin resistance, and dyslipidemia) who is taking a statin and has an LDL-cholesterol (LDL-C) level of 70 mg/dL but also has elevated triglycerides (TGs; 200 mg/dL) and low HDL-cholesterol (HDL-C; 30 mg/dL). What could be approaches to reduce atherosclerotic cardiovascular disease (CVD) risk in this patient? Approaches could first include further efforts to reduce LDL-C by maximizing the dose of potent statins, adding the cholesterol absorption inhibitor ezetimibe, or suppressing the LDL-R–regulating protein PCSK9 with monoclonal antibodies. While each of these treatments reduces CVD risk, they do not very consistently reduce TG levels and they still leave a substantial residue of CVD events (1–3). Treatment options for further lowering TGs might include fibrates or fish oils; however, the evidence for a beneficial effect of fibrates is not compelling (4), and while some fish oils may reduce CVD (5), the underlying mechanisms and impact remain uncertain. Although low HDL-C is associated with increased CVD risk, there are currently no effective drugs for targeting low HDL, and the whole idea of raising HDL-C has been called into question (6). Thus, beyond LDL-C, there are no optimal current treatment options to address dyslipidemia, as exemplified by this typical patient with metabolic syndrome.

**Figure 1:**
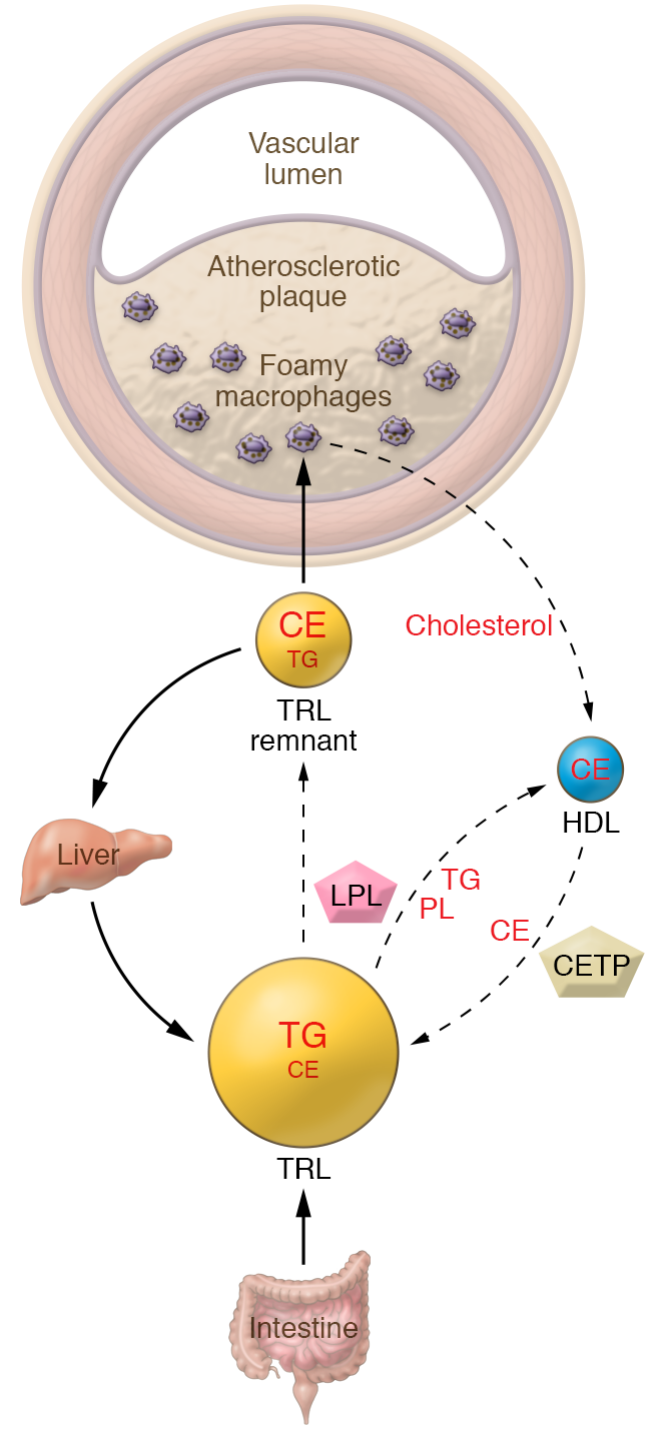
Lipoprotein modulation of atherosclerosis, beyond LDL. Circulating lipoproteins other than LDL modulate the development of atherosclerosis. Animal and human data show that cholesteryl ester–rich lipoproteins derived from the partial catabolism of TRLs, referred to as remnants, are taken up by macrophage foam cells in arteries, promoting development of atherosclerotic plaques. The catabolism of TRLs also mediates enrichment of HDL with phospholipids, increasing their ability to promote efflux of cholesterol from foam cells and thus ameliorating atherosclerosis. The interchange of lipids between HDL and TRL is mediated by cholesteryl ester transfer protein (CETP) and phospholipid transfer protein (not shown). CE, cholesteryl ester; LPL, lipoprotein lipase; PL, phospholipids.

A major theme of this Review is that lowering levels of TG-rich lipoproteins (TRLs) by activation of lipolysis and enhanced hepatic clearance of cholesterol-rich TRL remnants is likely to be beneficial for CVD (Figure 1). Activation of lipolysis leads to reduction in the levels of atherogenic TRL as well as increased levels of HDL: both effects may reduce atherosclerosis. While diet, exercise, and weight loss can have an important role in lowering TGs and raising HDL-C (7–10), the emphasis here will be on treatments using novel technologies to target new pathways that have been uncovered through genetic studies.

## Trends in residual risk

CVD, including myocardial infarction and stroke, is the leading cause of death in the United States and accounts for 28% of overall mortality (11). CVD risk factors include age, sex, hypertension, diabetes, smoking, body mass index, and increased levels of LDL-C or TGs or reduced HDL-C levels (1, 12). Current therapies for prevention of ischemic events focus on controlling risk factors, as well as suppressing thrombosis in at-risk individuals, and this approach has contributed to decades of improvement in CVD mortality (13). This trend has stalled in recent years, in part because of the rise of obesity, diabetes, and their associated dyslipidemias. The metabolic syndrome has become more prevalent in all sociodemographic groups in the United States, and now occurs in more than one-third of adults (14).

Both fasting and nonfasting TG levels associate with CVD risk. In a European population, 27% of adults had nonfasting TGs greater than 176 mg/dL, a level that was associated with an approximately 1.9-fold increase in risk of CVD (15). In individuals with TGs greater than 580 mg/dL, the risk was increased even further to 5.1-fold for myocardial infarction and 3.2-fold for ischemic stroke in comparison with subjects with low TG levels. The calculated remnant cholesterol level (total cholesterol minus LDL-C minus HDL-C) appears to provide comparable information on risk. Statins can moderately lower fasting and postprandial TG levels in hypertriglyceridemic patients (16), likely via enhanced hepatic LDL receptor–mediated (LDLR-mediated) remnant clearance. TG lowering is associated with reduced CVD risk in statin trials (1, 17), and elevated TG levels associate with residual risk in statin-treated patients, particularly in those with diabetes (18). Moreover, marked reductions in non-HDL cholesterol and to a lesser extent TGs associate with plaque regression as determined by intravascular ultrasound of coronary arteries (19). Several animal studies suggest that large minimally metabolized TRLs have reduced atherogenic potential compared with smaller TRL remnants or LDL. Such particles become the major circulating lipoproteins when cholesterol-fed rabbits are made diabetic with alloxan, but the particles penetrate the artery relatively poorly, and diabetic rabbits have less atherosclerosis than nondiabetic rabbits with smaller, more penetrant lipoproteins (20). Deletion of either lipoprotein lipase (LPL; ref. 21) or glycosylphosphatidylinositol-anchored HDL-binding protein 1 (GPIHBP1; ref. 22) causes severe hypertriglyceridemia, but mice only develop early atherosclerotic lesions at an advanced age. In mouse models of atherosclerosis regression, conditional knockout of LPL induced hypertriglyceridemia, reflecting increased nascent TRL but not VLDL-C or remnants, and did not affect atherosclerotic burden or morphology (23). Overall these observations suggest that while lowering TRL or more specifically remnant cholesterol levels is likely to reduce coronary atherosclerosis and CVD, many patients are inadequately treated by current medications, highlighting the need for new approaches to reduce TRL remnant levels.

## Insights from lipid trait genetics

While observational studies have linked HDL-C and TGs to CVD risk (24), such studies have a limited role in elucidating the causal risk conferred by these lipid fractions because of confounding by correlated metabolic syndrome traits and artifacts introduced by differential variability of these analytes (25). Human genetic variation in lipid trait–modifying genes has provided key insights into mechanisms and therapeutic opportunities in atherosclerosis. Rare mutations increasing LDL-C are consistently associated with increased CVD risk proportionate to the level of LDL-C elevation, and the study of common variants in LDL-C–modifying genes recapitulates this observation (26, 27). In contrast, genetic associations of HDL-C and TGs with CVD risk based on rare variants do not unambiguously identify the culprit lipoprotein, in part because of concurrent effects on multiple lipoprotein traits. With the availability of large genome-wide association studies of lipid traits and CVD, common variants in HDL-C– and TG-modifying genes have been used to model the causal effects of changes in HDL-C and TGs on CVD through the Mendelian randomization approach (28). Since the genotype is randomly allocated in the population being studied, it can be inferred that the CVD effect derives from either the lipid trait effect or cosegregating pleiotropic effects of the genetic variant. Multiple variants can be simultaneously analyzed by meta-analysis to increase power and reduce bias conferred by isolated pleiotropic effects of single variants (29–31).

Early Mendelian randomization studies confirmed the role of LDL-C in CVD and suggested that levels of TGs, but not HDL-C, causally contribute to CVD risk (30, 32). Subsequently, the application of meta-analysis in Mendelian randomization, which accounted for differences in measurement error between variants, suggested causal effects of both HDL-C and TGs (31, 33). Further analyses using methods robust to pleiotropy, e.g., Egger regression, called into question the causal effects of HDL-C and TGs in CVD (31, 34). The TG effects on CVD were attenuated by adjustment for apolipoprotein B (APOB) levels, suggesting that TG levels are a biomarker for a variable summarized by APOB levels, e.g., the cholesterol content of TRL (35). Studies conducted in the Icelandic population also suggested that the deleterious effect of TG-raising genetic variants is accounted for by the non–HDL-C lipid fraction and mediated by the atherogenic effect of TRL-C (36, 37).

Recently, our multivariable analysis using very large data sets and adjustment for widespread pleiotropic effects of lipid trait variants on metabolic syndrome traits revealed independent causal effects of HDL-C and TGs on CVD albeit of smaller magnitude than the well-known LDL-C effect (38). Our finding that LDL-C, HDL-C, and TGs are independently associated with coronary artery disease (CAD) by Mendelian randomization (38) builds on three recent reports showing independent Mendelian randomization associations of LDL-C, HDL-C, and TGs with abdominal aortic aneurysm (34, 39, 40), suggesting that each of these factors is biologically active at the level of the arterial wall. Our study suggested that some, but not all, mechanisms of HDL-C raising and TG lowering are associated with protective CAD effects. These mechanism-specific effects may have been missed in prior studies because of both effect heterogeneity and statistical power limitations related to sample size, which were magnified by the intrinsic power limitations of pleiotropy-adjusted Mendelian randomization techniques (41), but are concordant with the results of other recent studies in large cohorts identifying a causal CAD effect for subsets of HDL-C variants (42–44). HDL-C–raising variants in genes such as *LIPG* (encoding endothelial lipase) that regulate HDL catabolism and may have downstream effects on macrophage cholesterol efflux had causal protective effects on CVD (38). LIPG activity leads to catabolism of HDL phospholipids and may cause the resulting phospholipid-poor HDL to be a poorer cholesterol acceptor (45, 46). The rs77960347 SNP in LIPG encoding the N396S mutation confers a large increase in HDL-C. In Mendelian randomization based on CAD data from 20,913 CAD cases and 95,407 controls, a null association of LIPG N396S with CAD was reported (32). In a subsequent CAD genome-wide association study comprising 60,801 CAD cases and 123,504 controls (47), LIPG N396S was associated with a possible CAD effect with an odds ratio of 0.90 (*P =* 0.05). In our recent CAD meta-analysis of 122,733 CAD cases and 424,528 controls, LIPG N396S was associated with a CAD effect with an odds ratio of 0.90 (95% CI 0.86–0.95; *P =* 8.8 × 10^–5^) (38). Consequently, univariable Mendelian randomization on this single SNP reveals a causal protective effect of increased HDL-C conferred by LIPG activity. In contrast, HDL-C–raising variants in genes that regulate the cholesteryl ester content of HDL particles, such as *LCAT* (encoding lecithin-cholesterol acetyltransferase), actually increase CVD risk. Likewise, TG-raising variants in genes that regulate the catabolism of TRLs such as *LPL* or *ANGPTL4* increased CVD risk, whereas TG-raising variants in genes that regulate hepatic TG biosynthesis such as *MLXIPL* (encoding carbohydrate-responsive element–binding protein [ChREBP]) or *FADS1* (encoding fatty acid desaturase 1) had no causal effect on CVD. Thus, while our meta-analysis adjusted for pleiotropy showed overall causal effects of TG and HDL-C levels on CAD, Mendelian randomization at loci with well-established direct effects on HDL-C and TG levels indicates locus- and mechanism-specific causal effects of these factors on CAD (38).

## Recent developments in understanding of TRL metabolism

**Figure 2:**
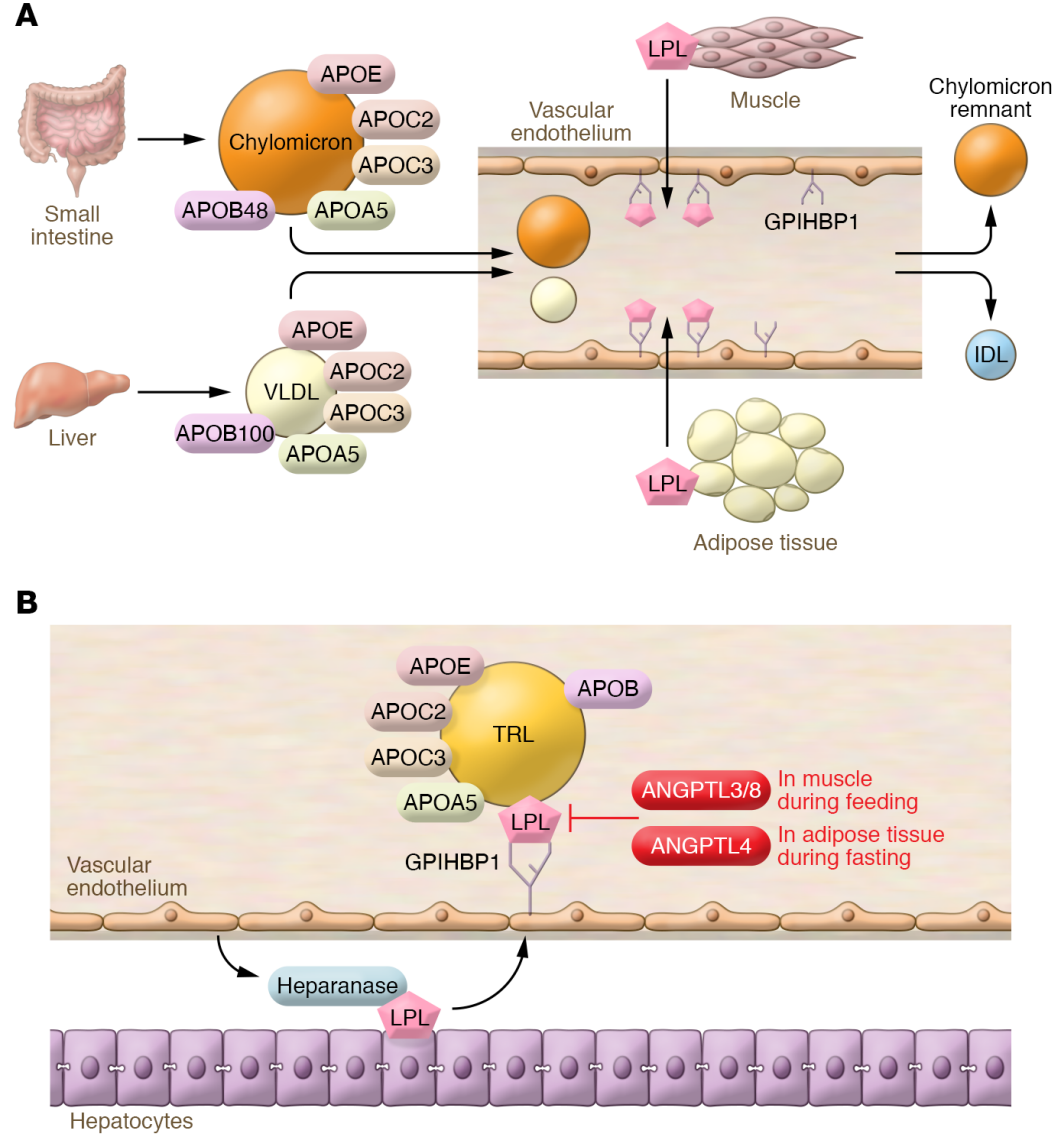
Lipolysis and TRL metabolism. (**A**) Lipolysis of circulating TRLs. Chylomicrons assembled in the small intestine and VLDL assembled in the liver contain proteins that control their intravascular metabolism. APOC2 is the activator of LPL. APOA5 also acts to enhance lipolysis, while APOC3 inhibits lipolysis. LPL is predominantly synthesized in adipose tissue, skeletal muscle, and heart. LPL transfers to the capillary lumen, where it associates with glycosylphosphatidylinositol-anchored HDL-binding protein 1 (GPIHBP1), releases free fatty acids from TRLs, and creates chylomicron remnants and intermediate-density lipoproteins (IDLs). (**B**) Lipolysis reaction. TRLs associate with LPL in the capillary lumen, a process thought to be assisted by APOA5. APOC2 activates LPL; APOC3 inhibits LPL. ANGPTLs also inhibit LPL. ANGPTL3, primarily produced in the liver, is most active in complex with ANGPTL8. ANGPTL4, though widely expressed, modulates LPL activity especially in adipose tissue.

TRLs transfer TGs from the diet or liver to tissues such as muscle and adipose for energy or fuel storage. Chylomicrons and VLDL require a lipolysis step to break the ester bond between fatty acids and glycerol (Figure 2A); luminal endothelial cell–associated LPL mediates this reaction. LPL is a member of a gene family that includes hepatic lipase and LIPG; the primary substrates of the latter enzymes are remnant lipoproteins and HDL. LPL binds to GPIHBP1 (48), which protects it from inactivation by angiopoietin-like 4 (ANGPTL4) (Figure 2B and ref. 49). Activity of LPL is also modulated by fatty acids that inhibit its catalytic activity and release it from endothelial cells.

The protein cargo of circulating TRLs regulates their intravascular metabolism (Figure 2A). The APOC proteins are components of TRLs and HDL and transfer from HDL to TRL in the postprandial state (50). APOC2 is the obligate activator of LPL, and APOC2 deficiency leads to familial chylomicronemia syndrome. A recent study has shown that an APOC2 analog that also reduces APOC3 increases lipolysis and reduces circulating TG levels (51). LPL activity is inhibited by APOC3, perhaps because it shields TGs or blocks association of LPL and APOC2. APOC3 also inhibits remnant clearance (see below). APOA5 deficiency leads to hypertriglyceridemia, which may reflect its role in the efficient association of TRL with LPL or GPIHBP1 on endothelial cells (52), possibly because APOA5 interferes with an inhibitory effect of ANGPTL3/ANGPTL8 on this association (53).

ANGPTL3 and ANGPTL4 bind to the C-terminal region of LPL, disrupting its tertiary structure and canceling its activity in a tissue-specific fashion (Figure 2B and ref. 49). ANGPTL4 is induced in adipose tissue during fasting, inhibiting the lipolytic uptake of fatty acids (54). ANGPTL3 is primarily synthesized in the liver and associates with circulating ANGPTL8 to enhance its LPL-inhibitory actions in muscle, thus acting in an endocrine fashion (55, 56). ANGPTL8 is also synthesized in adipose tissue and acts in a juxtacrine or paracrine fashion to block the inhibitory effect of ANGPTL4 on adipose LPL through formation of ANGPTL4/ANGPTL8 complexes (56, 57). ANGPTL8 is induced in both liver and adipose tissue in the postprandial state, thus leading to increased uptake of fatty acids in adipose tissue and reduced uptake in muscle (56, 57). The complex posttranslational regulation of LPL activity by ANGPTLs therefore controls the tissue distribution of TG-derived fatty acids during feeding and fasting.

Tissue uptake of lipids from chylomicrons differs from that of lipids from VLDL in part because of differences in the movement of lipoprotein lipids across the endothelial cell barrier. This might reflect the local concentrations of fatty acids, which likely are much greater after lipolysis of chylomicrons versus VLDL. Although endothelial cells can internalize and degrade TRLs in lysosomes (58, 59), uptake of TRL TGs primarily occurs after LPL generation of nonesterified fatty acids (NEFAs). Studies of the fatty acid transporter CD36 in endothelial cells (60) implicate this receptor in fatty acid uptake when NEFA levels are low (61). Therefore, CD36 might be most important during fasting or perhaps VLDL hydrolysis; with higher NEFA levels, the receptor plays a less important role. Consistent with this, chylomicron-derived NEFAs and cholesteryl esters do not require CD36 for their movement into the heart (62, 63), and likely either other organs use other receptors or chylomicron-derived NEFAs cross the endothelial barrier via a paracellular route. In contrast, uptake of VLDL-derived lipids into the heart (62) and brown adipose tissue (64) is reduced by CD36 deficiency.

## Potential mechanisms of benefit from lowering of TRLs

The cholesterol-rich remnants of chylomicrons or VLDL accumulate in the arterial intima, promoting macrophage foam cell formation and inflammatory changes in atherosclerotic plaques (Figure 1). However, as with LDL, it is not completely clear what are the most important toxic molecules in TRL remnants; candidates include cholesterol, phospholipids or their oxidation products, fatty acids derived from lipolysis, and immunogenic APOB. The strongest evidence suggests an atherogenic role of TRL remnant cholesterol (15). Cholesterol-rich remnant particles carry a bigger load of cholesterol, are more effective at inducing macrophage foam cells than LDL, and do not need to be modified by oxidation in order to be taken up by macrophages (18). Free fatty acids liberated during lipolysis of TRL on the endothelial surface also can exert proinflammatory effects on endothelial cells and macrophages (65, 66).

Despite the correlation of atherosclerosis risk with VLDL or LDL cholesterol, recent immune cell profiling of atherosclerotic plaques by single-cell RNA sequencing studies has introduced a conundrum: Trem2-high cholesteryl ester–laden foam cells have lower expression of inflammatory genes compared with other populations of macrophages in plaques (67). Glass and colleagues have shown that cholesterol-laden foam cells suppress inflammatory pathways as a result of accumulation of desmosterol, a ligand of liver X receptor (LXR) (68); LXR activation reduces expression of inflammatory genes by *cis*-repression and other mechanisms (69). Recent evidence also highlights the atherogenic role of lipoprotein-derived oxidized phospholipids (70). Together these studies suggest that oxidative or other modifications of remnant particles or LDL that produce danger-associated molecular patterns to activate Toll-like receptor signaling may be more important in inducing inflammatory responses than macrophage cholesterol loading per se. However, excessive cholesterol loading that is not compensated by cholesterol esterification or cholesterol efflux may lead to ER stress and cell death (71), as well as to activation of the NLRP3 inflammasome and formation of neutrophil extracellular traps (72, 73), contributing to the formation of unstable plaques, plaque erosion, and thrombosis (74).

## Dyslipidemia and vascular inflammation

Recent human CVD outcome trials using IL-1β antibodies (75) or colchicine (76) have demonstrated reduced CVD. This underlines the role of inflammation in the clinical complications of atherosclerosis and opens a new vista on antiinflammatory therapies as potential treatments for atherosclerotic CVD. However, these studies should not be viewed in isolation, because, as indicated above, the inflammatory component of atherosclerosis is intimately connected to the dyslipidemia that promotes the accumulation of atherogenic lipoproteins in the artery wall. Accordingly, elevated levels of remnant cholesterol are associated with higher CRP levels, a biomarker of inflammation (77). Moreover, dyslipidemia activates hematopoiesis and myelopoiesis, promoting formation of increased numbers of inflammatory monocytes and neutrophils that are poised to enter the artery wall and promote atherothrombosis (78–81). Thus, dyslipidemia contributes to the inflammatory risk of atherosclerosis. In some patients, vigorous control of dyslipidemia may adequately reduce inflammatory risk, obviating the need for potentially immunosuppressive antiinflammatory treatments. However, in others genetic inflammatory risk may be increased (e.g., as in clonal hematopoiesis of indeterminate potential; refs. 82–84) and control of dyslipidemia may need to be buttressed with antiinflammatory therapies. A recent study showed that antiinflammatory therapies that lowered CVD risk were associated with reductions in the neutrophil/lymphocyte ratio, a global marker of inflammation; however, lipid lowering with statins or PCSK9 inhibitors did not affect this ratio (85). Thus, targeting antiinflammatory therapies to susceptible populations identified by genetic risk factors or by appropriate biomarkers may be an important addition to controlling dyslipidemia.

## Emerging targets in dyslipidemia based on genetic studies

### APOC3.

**Figure 3:**
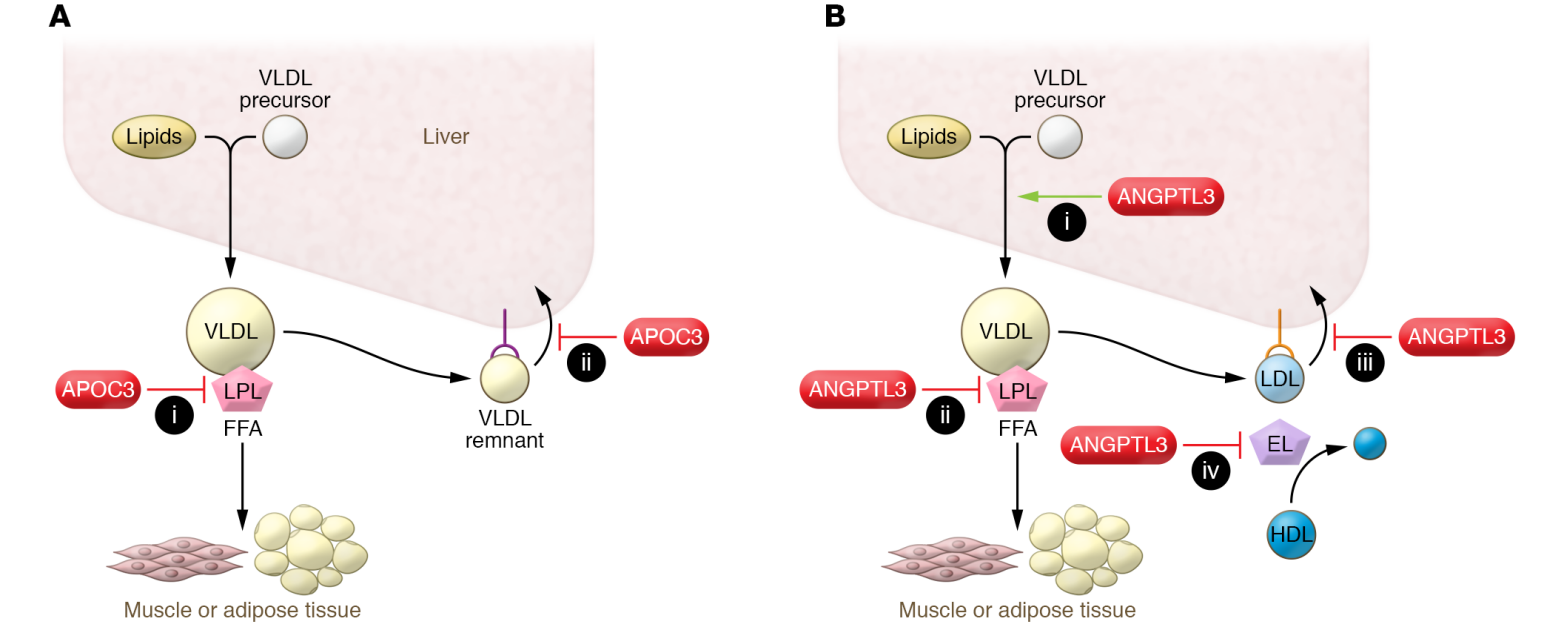
Two potential new therapies to reduce TRLs. (**A**) APOC3 depletion via knockdown in the liver or antibody inhibition in the circulation reduces circulating TG levels via two mechanisms: (i) removal of APOC3 releases its inhibition of LPL and increases intravascular lipolysis, and (ii) loss of APOC3 promotes uptake of TRL in the liver. (**B**) ANGPTL3 depletion reduces TRLs and LDL via (i) reduced liver TG secretion; (ii) increased intravascular lipolysis; and increased hepatic removal via either (iii) LDLR-dependent, non–endothelial lipase–dependent or (iv) non–LDLR-dependent, endothelial lipase–dependent processes. HDL levels decrease with ANGPTL3 loss as a result of activation of endothelial lipase. EL, endothelial lipase; FFA, free fatty acid.

It has long been known that plasma APOC3 concentrations correlate with TG levels (86), are elevated in patients with diabetes, and associate with increased coronary atherosclerosis and CVD risk (87, 88). The causal role of APOC3 in increased TG levels was first shown by overexpression of *APOC3* in transgenic mice (89), and the pro-atherogenic role of APOC3 was demonstrated in *APOC3*-transgenic, LDLR-deficient mice (90). Animal and cell studies suggest that APOC3 deficiency has two main TG-lowering effects: activation of LPL-mediated lipolysis and promotion of the hepatic clearance of TRL remnants (Figure 3A). APOC3 inhibits the hepatic clearance of TRL remnants via the LDLR and LDLR-related protein (LRP) (91). The effects of APOC3 inhibition on hepatocyte clearance of TRL remnants are not seen in the absence of APOE and likely reflect reduced masking of APOE by APOC3 rather than reduced displacement of APOE (92). However, APOC3 deficiency or inhibition lowers TG levels in the absence of APOE likely as result of increased LPL activity and uptake of fatty acids in adipose tissue (92).

The translational importance of these studies in animals and cells was illuminated by human genetic findings. Shuldiner and colleagues (93) showed that loss-of-function *APOC3* variants in the Amish associated with reduced TG levels and decreased coronary atherosclerosis. These findings were extended in large population studies in the National Heart, Lung, and Blood Institute Exome Sequencing Project (94) and in two other European population studies (95). Four different *APOC3* variants were found: three nonsense mutations and one missense mutation with an overall frequency of 1 in 150. In heterozygotes, there was a 39% lowering of TGs, a 46% lowering of APOC3 levels, a 25% increase in HDL-C, and a 40% reduction in CVD risk (94). In a smaller study in the Icelandic population, the most common *APOC3* loss-of-function variant did not associate significantly with CVD, consistent with a more modest effect size (36). Homozygous *APOC3* deficiency is associated with markedly reduced fasting and postprandial TG levels and appears not to have adverse health effects (96).

The impact of heterozygous loss-of-function *APOC3* mutations on LDL-C is minimal (–4%) (95), suggesting the importance of lowered remnant cholesterol and possibly increased HDL in the beneficial effects of APOC3 deficiency. Lipoprotein turnover studies in small groups of subjects with homozygous (93) or heterozygous (97) deficiencies of *APOC3* showed increased fractional clearance of VLDL TG and APOB, and increased conversion of VLDL to LDL without clearly increased hepatic clearance of remnants. However, in large population studies, APOC3 deficiency was associated with reduced VLDL and non-HDL cholesterol levels that appeared to be largely responsible for the CVD benefit (98). APOC3 might have additional atherogenic effects. LDL enriched with APOC3 associates strongly with CVD risk (99), perhaps because APOC3 increases LDL affinity for proteoglycans (100), and as mentioned above, APOC3 may increase vascular inflammation.

An antisense oligonucleotide (ASO) to *APOC3* (volanesorsen) very effectively lowered TG levels in patients with familial chylomicronemia syndrome (FCS), including those with LPL-null mutations (101). Volanesorsen lowered TG levels in patients with moderately severe hypertriglyceridemia (mean TGs = 580 mg/dL), but also resulted in significant LDL-C elevations and no overall change in non-HDL cholesterol or APOB levels (102). Volanesorsen treatment was subsequently found to induce clinically significant thrombocytopenia in a few patients with FCS (103). The use of hepatocyte-targeted ASOs to lower APOC3 is still under investigation as a therapeutic for FCS and recurrent pancreatitis (101). RNAi approaches have recently been introduced to reduce hepatic PCSK9, producing sustained LDL lowering after only two doses (104). RNAi lowering of APOC3 has also been reported with potent TG and APOC3 lowering, increased HDL-C, and prolonged effects of more than 10 weeks (105). Monoclonal antibody approaches to APOC3 lowering have also been described (106). A potential advantage of the antibody approach is their ability to inhibit APOC3 produced both in the liver and in the intestine, while a challenge is that APOC3 is an abundant plasma protein.

### ANGPTL3.

*ANGPTL3* deficiency causes pan-hypolipidemia (107) and reduces CVD risk (108). Relative to individuals with functional ANGPTL3, subjects with various rare heterozygous loss-of-function mutations had mean TGs decreased by 27%, LDL-C by 9%, and HDL-C by 4% with an odds ratio for CVD of 0.61. ANGPTL3 antibodies (evinacumab) and ASOs (vupanorsen) (109) are being developed as potential treatments of CVD. ANGPTL3 antibodies lower LDL-C independently from LDLR activity, and, notably, evinacumab lowers LDL-C by about 50% in familial hypercholesterolemia homozygotes (110). In addition to inhibiting LPL, ANGPTL3 also inhibits activity of LIPG, and this accounts for the HDL-lowering effect of ANGPTL3 inhibition (111, 112). Two recent studies (113, 114) have shown that in the absence of LDLRs, VLDL/LDL-lowering effects of ANGPTL3 inhibition are mediated through increased LIPG activity. However, ANGPTL3 inhibition does lower LDL-C in *Lipg–/–* mice lacking LIPG but with functioning LDLR (113). Thus, ANGPTL3 reduction appears to increase two pathways of LDL-C reduction, one via the LDLR independent of LIPG and a second requiring LIPG but not the LDLR (Figure 3B).

An *LIPG* reduced-function variant that increases HDL-C and to a much lesser extent non-HDL cholesterol is associated with reduced CVD (38). *Lipg–/–* mouse models have shown either reduced (115) or unchanged atherosclerosis (116).Together these studies suggest that in the presence of functioning LDLR the benefit of ANGPTL3 inhibition may be attenuated by increased LIPG activity as a result of reduced HDL-C and reduced cholesterol efflux capacity (117). While evinacumab is being developed for treatment of familial hypercholesterolemia, the potential for wider application in dyslipidemia could be limited by HDL-C–lowering effects. A beneficial effect of ANGPTL3 inhibition on insulin resistance and fatty liver has been suggested (109, 118); however, liver-targeted antisense inhibition of ANGPTL3 with vupanorsen in subjects with fasting hypertriglyceridemia and increased liver fat, while lowering TG levels by about 60%, did not result in reduced steatosis or improved glycemic parameters (119).

### ANGPTL4.

Reduced-function variants of *ANGPTL4* have been associated with lower TG levels, increased HDL-C, and reduced CVD risk (120). This suggested that, like targeting of ANGTPL3, targeting of ANGPTL4 could lower TGs and CVD risk. However, in mice, homozygous *Angptl4* deficiency resulted in lipogranulomatous lesions of the intestines and their draining lymphatics, probably reflecting premature lipolysis of chylomicrons and macrophage inflammation (121, 122). These adverse effects, although not reported in humans, appear to have impeded the clinical development of ANGPTL4 inhibitors. However, deletion of ANGPTL4 in hepatocytes resulted in reduced TG levels and protected against diet-induced obesity, glucose intolerance, liver steatosis, and atherosclerosis, without the previously described complications of whole-body *Angptl4* deficiency (123).

### Fish oils (n-3 long-chain polyunsaturated fatty acids).

Marine oils lower plasma TG levels in humans and have been extensively assessed for potential CVD benefit. The TG-lowering mechanism of *n*-3 fatty acids is related to decreased hepatic VLDL production (124). Four grams per day of icosapent ethyl (Vascepa, an ester of eicosapentaenoic acid [EPA]) substantially reduced TG levels and CVD events in statin-treated patients with elevated TGs (135 to 499 mg/dL) in the REDUCE-IT trial (5); this is reminiscent of the results of an earlier open-label trial using 1.8 g/d EPA in hypercholesterolemic Japanese patients (125, 126). TG lowering is unlikely to be the full explanation for the CVD benefit of icosapent ethyl, as benefits were similar irrespective of the degree of TG lowering in subjects receiving icosapent ethyl. In contrast, a lower dose of 1 g combined EPA/docosahexaenoic acid (DHA) failed to show a CVD benefit in diabetics (127), and 4 g/d of a mixture of EPA/DHA versus corn oil failed to show a benefit in statin-treated subjects with high CVD risk in the STRENGTH trial (128). The apparent CVD benefit of icosapent ethyl may be related to the dose of EPA, differences between EPA and DHA, the targeting of a susceptible population with elevated TGs over 150 mg/dL, and the long duration of the trial (5 years). However, concerns have been raised regarding the use of mineral oil as a control, which could potentially have decreased statin absorption, as control subjects experienced an increase in LDL-C and CRP (129). Modeling of the predicted CVD effects of observed plasma lipid and CRP changes resulting from administration of mineral oil or EPA suggests that a considerable part of the apparent CVD benefit in REDUCE-IT was related to adverse effects of mineral oil in the control group, while about 13% of the CVD benefit was related to effects of EPA or mineral oil not mediated by lipid and CRP changes (124, 130–132). Increased formation of inflammation resolution mediators could be part of the benefit derived from EPA; failed inflammation resolution can impair regression of atherosclerosis (133). Further studies on fish oils may help to provide a clearer understanding of the mechanisms and magnitude of their CVD benefit.

## Challenges in the development of new TG-lowering agents

In contrast to LDL lowering, it is unlikely that all modes of TG lowering will have similar impact on CVD. Our Mendelian randomization studies suggest that genetic variants that cause activation of lipolysis may have beneficial effects on CVD, while some variants that decrease VLDL production do not associate with reduced CVD (38). Decreasing VLDL production, e.g., by APOB ASO treatment, may also lead to nonalcoholic fatty liver disease (134). Fibrates lower TG levels by decreasing APOC3 and increasing lipolysis; however, fibrates have pleiotropic effects, and the lack of clear-cut benefits has impeded their acceptance. A new class of fibrates is being evaluated in patients with high TGs and low HDL (135).

The amount of TG lowering in clinical trials that is likely to produce benefit is unclear. As with LDL-C, the reduction in CVD risk from TG or remnant-cholesterol lowering appears to be proportional to the absolute reduction in TRL levels, and thus for a given percentage reduction in TGs, the impact will be larger when baseline TG levels are higher (17, 136). This emphasizes the importance in clinical trials of targeting populations with substantial elevation in TG levels at baseline, as was done in the more successful trials of fibrates (4, 136) and of icosapent ethyl (135).

## Potential benefits of increasing reverse cholesterol transport

Activation of lipolysis of TRL leads to an increase in HDL levels as a result of the transfer of surface phospholipids and APOA1 from TRL into HDL as well as reduced cholesteryl ester transfer protein–mediated (CETP-mediated) exchange of TRL TG for HDL cholesteryl ester (Figure 1). The enrichment of HDL with phospholipids and APOA1 is likely to increase its ability to promote efflux of cholesterol from macrophages and endothelial cells in atheromatous plaques via ABCA1 and ABCG1 transporter–dependent and other cholesterol efflux pathways (137, 138). A number of studies indicate that the ability of HDL to promote efflux of cholesterol from macrophages is inversely correlated with CVD risk (139). Although cholesterol efflux is correlated with HDL-C levels, multivariate analysis has shown a strong effect of cholesterol efflux on CVD independent of HDL-C levels. Higher levels of cholesterol efflux capacity may be related to an increased content of phospholipids in HDL (140), which could also explain the apparent CVD benefit of the LIPG N396S reduced-function variant (38). The cholesterol mass efflux capacity of HDL shows an inverse relationship to coronary artery disease but not to thrombotic stroke (141) or peripheral artery disease (142), indicating a differential effect of HDL-mediated macrophage cholesterol efflux in different vascular beds. A recent study has shown that the ability of HDL to suppress inflammatory responses in cultured endothelial cells is inversely correlated with incident CVD and apparently independent of macrophage cholesterol efflux capacity (143).

LXR activators appear to be ideal drugs for increasing reverse cholesterol transport and suppressing plaque inflammation (144). They are potently and consistently anti-atherogenic in animal models. However, clinical development in humans was halted because of adverse effects on hepatic steatosis and liver function tests and an increase in LDL-C levels that likely reflects a mechanism-related suppression of hepatic LDLR (145, 146). As an alternative, plaque macrophage–targeted nanoparticles containing LXR activators might represent an attractive option for LXR therapeutics (147).

## HDL-directed therapeutics

The results of clinical trials of agents that raise HDL-C have been disappointing (6). In particular, CETP inhibitors markedly increased HDL-C levels and moderately reduced LDL-C levels. While the largest and longest trial of CETP inhibition using anacetrapib showed a highly significant 9% reduction in CVD endpoints (148), anacetrapib has not been marketed for CVD, probably owing to the moderate effect size and the long-term accumulation of the drug in adipose tissue. Moreover, this CVD reduction might primarily reflect the LDL-C reduction that occurred (149). HDL infusions and overexpression of *APOA1* in animal models consistently reduce atherosclerosis (150–152), suggesting a direct anti-atherogenic action. This has led to clinical trials of infused reconstituted HDL (rHDL) particles consisting of phospholipids and APOA1. In animals, such preparations have anti-atherogenic (153) and antiinflammatory effects (154). Although proinflammatory effects of such preparations have been described, these largely occurred under conditions of extreme cholesterol depletion as a result of an ER stress response and do not occur in atherosclerotic plaques (154).

Infusions of early formulations of rHDL such as CSL-111 improved the characteristics of coronary artery plaques in imaging studies but also caused significant elevation of liver function tests (155). A new formulation of rHDL, CSL-112, containing reduced phospholipids relative to APOA1, produced lower levels of cholesterol efflux but did not induce elevations in alanine transaminase (a potential biomarker of liver injury) when infused into mice or humans (156–158). Four infusions of CSL-112 are currently being assessed as a treatment to prevent recurrent CVD in patients with severe coronary disease. Although this study represents a direct test of the anti-atherogenicity of HDL in humans, it remains to be seen whether the dose and duration of rHDL treatment will be sufficient to reduce CVD.

## Summary and perspective

Goldstein et al. reported in this journal more than 40 years ago that hypertriglyceridemia was commonly found in patients with coronary artery disease (159). Zilversmit proposed that cholesterol-rich remnants of TRL might be atherogenic (160), and over the last 40 years epidemiological and genetic evidence has accrued to support the hypothesis that remnants increase CVD risk, while reduction of remnant levels by statins or other treatments may be beneficial. However, we still are uncertain as to the optimal lipid-modifying therapies for targeting TGs in the 60-year-old metabolic syndrome patient discussed in the introduction. There is hope that a new class of therapeutics based on rare mutations affecting TG levels will activate lipolysis, promote remnant clearance, and reduce CVD. To determine whether these new therapies reduce CVD will require large clinical outcome studies. That any CVD benefit derives directly from lowering TG or remnants is unlikely to be revealed because, like fibrates and fish oils before them, these newer TG-reducing medicines will have multiple effects on lipoproteins and other atherosclerosis risk factors. Nonetheless, while as a research community we continue to work toward a clearer understanding of the complicated biology associated with circulating lipoproteins and CVD, based on new approaches to lowering TRL and increasing HDL-C, clinical medicine will likely advance toward improved patient outcomes.

## Version 1 01/04/2022

Electronic publication
